# Camphorated Chloroform Liniment

**Published:** 1849-08

**Authors:** 


*Camphorated Chloroform Liniment.*—A communication from the London Lancet, published in the Eclectic department of our last number, gives a formula for preparing a mixture of Chloroform and Camphor, held in solution by the yolk of an egg; thus procuring a very elegant preparation by which a larger dose of camphor can be administered, than by any other known method. We have tried this remedy in several instances, and have found it to afford relief in an obstinate case of flatulent colic, and in several cases of Dysmenorrhœa. In the latter complaint we believe it to be a valuable remedial agent; and we refer to the fact of the powerful solvent properties of chloroform, to introduce to the notice of our readers, a liniment that has been prepared by William B. Price, an enterprising druggist of this city, where olive oil is used as the vehicle of the compound of camphor and chloroform, instead of the egg, as mentioned in the communication referred to. The officinal Linimentum Camphoræ of the U. S. Dispensatory contains half an ounce of camphor to two fluid ounces of olive oil, but it has been ascertained by the gentleman referred to, that by the aid of chloroform a much larger quantity of camphor may be held in solution, thus increasing greatly the strength of the compound. As three drachms of camphor are perfectly soluble in one fluid drachm of chloroform it is clear that the strength of the officinal liniment may be greatly increased by the use of the latter solvent. The present formula gives us half an ounce of camphor to two fluid ounces of oil; by dissolving the camphor in chloroform, it may be increased in weight to one ounce and a half, and will be held in solution by the same quantity of oil, with the addition of two fluid drachms of chloroform, to give an anesthetic property to the liniment. We have used this liniment in a few cases of local pain from neuralgia and rheumatism with good effect. Our patients speak of it as a soothing and pleasant remedy;—it is worth a trial.—*New Jersey Medical Reporter.*

